# Sensory Profile and Consumer Liking of Sustainable Salamis Differing in Wild Boar Meat and Seasoning Ingredients Addition

**DOI:** 10.3390/foods12051089

**Published:** 2023-03-03

**Authors:** Pierangelo Freschi, Ada Braghieri, Corrado Pacelli, Emilia Langella, Amelia Maria Riviezzi, Rosanna Paolino, Carlo Cosentino

**Affiliations:** School of Agricultural, Forestry, Food and Environmental Sciences (SAFE), University of Basilicata, 85100 Potenza, Italy

**Keywords:** cacciatore salami, wild boar meat, sensory properties, consumer liking

## Abstract

The production of game meat is a proven way of promoting sustainable food, which is also consistent with the proper management of the expansion of the wild boar population in Italy. In the present study, we investigated consumer response to sensory attributes and consumer preference for ten types of “cacciatore” salamis prepared with different mixtures of wild boar/pork (30/50 or 50/50) and spice ingredients. PCA analysis showed a clear characterization of the salamis based on the first component with the hot pepper powder and fennel types differing from the others. For the second component, salamis without flavorings could be discriminated by those flavored with aromatized garlic wine or with black pepper only. The main findings of the hedonic test revealed that products with hot pepper and fennel seeds received the highest ratings, as well as satisfactory acceptance in the consumer test sensory analysis for eight out of ten products. The panelists and consumers’ ratings were influenced by the flavors used, but not by the ratio of wild boar to pork. This gives us the opportunity to produce more cost-effective and environmentally friendly products, as doughs with a high proportion of wild boar meat can be used without affecting product preference.

## 1. Introduction

The increase in harvested wild boar from less than 50,000 in the mid-1980s to more than 300,000 in recent times indicates the recent and significant demographic spread of wild boar throughout Italy [1]; on a global scale, the rapid and extensive global spread has made this species one of the 100 worst invasive animals and pests [2]. Damage currently attributed to wild boar includes: agricultural crops, rangelands, commercial woodlands; predation and disease transmission to livestock such as African swine fever [3] and pseudorabies [4]; environmental damage to native plant and wildlife species; wetlands and water quality in surface waters; green spaces (e.g., parks, suburban/urban landscapes); threats to human health [5], including infectious diseases caused by bacterial pathogens (e.g., *Yersinia enterocolitica*, *Brucella suis*, *Salmonella* spp., *Leptospira* spp., and *Escherichia coli*), endoparassitosis (e.g., *Trichinella* spp.), and collisions between wild boars and vehicles resulting in property damage, human injuries, and death [6,7]. The application of culling plans to limit the damage of this species is leading to widespread availability of wild boar meat in many developed countries [8,9], resulting in an increased supply of processed products. Although a market for processed game meat from hunting has been established in France and Spain [10], game meat in Italy is primarily consumed by hunters and their families, and the lack of a structured food supply chain limits the distribution of these products to a few regions in central northern Italy, mainly Umbria and Tuscany [11]. On the other hand, consumer interest in game meat, which is considered more “natural” and with excellent sensory and nutritional properties, is growing. Wild game meat is considered more “natural” as wild animals are not exposed to the stress associated with industrial breeding [11,12] and, when properly hunted, do not have the stress of transport to the slaughterhouse [13]. In addition, the use of game in meat products would also be more sustainable than the use of pure pork. The use of a fine grain of the dough and of flavorings could mitigate the organoleptic differences in a very heterogeneous raw material, mainly due to the different age and quality of the carcasses of the hunted animals. Furthermore, the guidelines recently issued in Italy for the hygiene of wild boar meat [14] will certainly allay the food safety concerns of unsettled consumers, while also creating supply chains for wild boar meat in the context of its wide availability and increasing consumer demand [12]. In light of this last point, and given the importance of consumer attitudes and purchasing behavior for various types of food preparation [15,16,17,18,19] and for wild boar meat in particular [13,20,21,22,23], in the present study we investigated consumer response to sensory attributes and consumer preference for ten types of “cacciatore” salamis, prepared with various blends of wild boar, pork meat and spice ingredients.

## 2. Materials and Methods

The study was carried out in the Basilicata region, where 7,225 wild boars were culled in the 2017 hunting season [24] and which had a population of about 89,000 wild boars at last census. This region has a long tradition of producing cured meat as testified by Marco Gavio Apicius, between 25 BC and AD 37, who wrote down the recipe for the oldest lucanian salami in the second book of *De re coquinaria*, which became known as “lucanica”. In this study we utilized a “Cacciatore” type salami, one of the most widespread types of salami in Italy, made from pork and various adipose components and spices, which originated in northern Italy during the Longobard invasions.

### 2.1. Raw Material

The processing of wild boar meat began in the first ten days of November with the culling of four adult wild boars in the Appennino Lucano–Val d’Agri–Lagonegrese National Park: two males weighing 60 and 65 kg and two females weighing 50, and 53 kg. The animals were culled by selection hunters using the waiting method. This hunting strategy is distinguished by remarkably low *ante mortem* stress for the animals, as the hunters remain stationary in a predetermined location and wait for the animal to approach them [1]. The carcasses were then sent to the cutting laboratory after a veterinary inspection and trichinoscopic examination. The pork cuts from a commercial hybrid line were supplied by the same plant where the raw meat was processed, stuffed, and then seasoned.

Our research does not fall within Directive 63/210 of the European Parliament and of the Council on the protection of animals used for experimental purposes (transposed into Italian law by Legislative Decree 26/2014) and, thus, it does not require any authorization from the national competent authorities. The protocol code of the certification of our Ethics Committee is OpBA 05_2023_UNIBAS.

### 2.2. Product Processing

Sausage making was performed according to a randomized block design with ten batches and three replicates. After rapid cooling at 0/2 °C, the wild boar meat obtained from the whole carcasses was put into the mincer and cut into pieces of the typical size (3–4 mm) of a “Cacciatore” salami. The pork cuts used (bacon and shoulder) were minced separately, in the same way as the wild boar meat. Ten different mixtures were made from the three sources of minced meat (wild boar meat, pork belly, and p rk shoulder), which differed in the ratio of wild boar to pork and in the seasoning ingredients added (i.e., garlic-flavored “Aglianico del Vulture” red wine, hot pepper, fennel seeds, black pepper, and wine), as depicted in Table 1. The garlic-flavored wine was obtained by steeping three garlic cloves, cut lengthwise, for 24 h per liter of wine. All the spices used in the experiment were commercial products.

**Figure A1:**
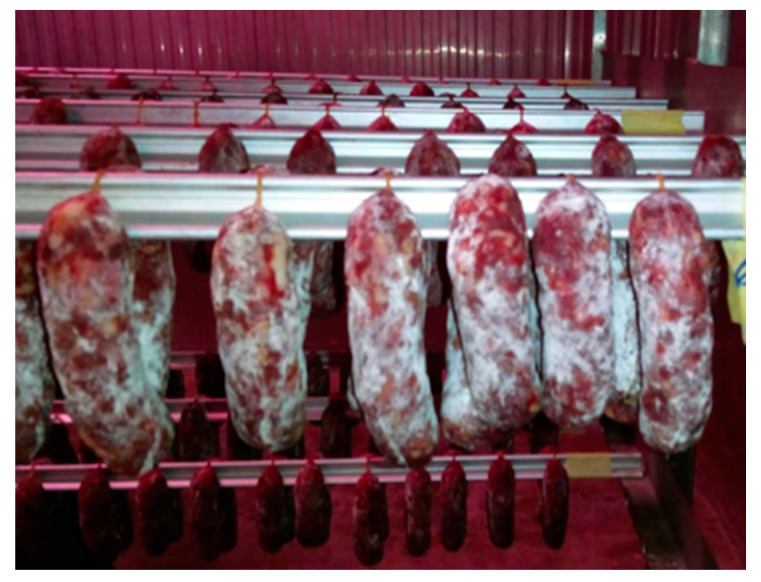
Salami in the last seasoning period.

The same amounts of NaCl (23 g/kg), NaNO_2_ (50 p.p.m.), fructose (3 g/kg), and lactic ferments (1 mL/kg) were added to the ten batches. Lactic ferments consisted of a mixture of *Lactobacillus sakei*, *Staphylococcus carnosus*, and *Staphylococcus xylosus* (Sacco Clerici Comp.). Each dough was immediately filled into natural casings with a diameter of about 4 cm. Hand tied every 10 cm, the casings were hung in pairs on aluminum rods (Figure A1). The salamis were then weighed and placed in a drying cell at 22 °C and 85% relative humidity. The weight loss percentage ranged from 38.27 (30-CF) to 45.96% (50-CF) (Table A1). Thirty-six hours after filling, the salamis were sprayed with a suspension of *Penicillium* spores, which resulted in the formation of white molds on the outside of the casing. The pH was measured inside the sausage on days 2, 4, 6 and at the end of ripening after 30 days (Table A2) using a portable pH meter HI931410 (Hanna Instruments, Woonsocket, RI) and a combined glass electrode. The apparatus was calibrated with 4.01 and 7.01 buffer solutions, according to the manufacturer’s methodology. The chemical, physical, and sensory properties of the cured meat were evaluated at the end of the curing phase (30 days at 16 °C and 65–70% RH).

### 2.3. Proximate Physical and Chemical Analyses

The sausages were weighed immediately after their preparation and successively after 10, 20, and 30 days, at the end of the maturing process. At the end of the curing phase (30 d), color was measured on three 0.4 mm slices of each batch using a Spectrophotometer CM-2600d (Minolta Co., Osaka, Japan), using the illuminant A and 10° observer in L*, a*, b* color space [25]. The chemical analyses were carried out with NIRS (Near Infrared Reflectance Spectroscopy, Foodscan Foss, Hillerød, Denmark) at the end of ripening on each sausage sample (Table A3).

### 2.4. Sensory Analises

#### 2.4.1. Panel Selection and Training

Fifteen subjects were recruited among regular eaters of sausages (defined as consuming the product at least once a week). Ten panelists were selected (four males and six females, between 29 and 61 yr. of age) in accordance with ISO standards [26]. For this purpose, the four basic tastes were used [27]. For this purpose, sucrose (Carlo Erba, Milan, Italy), sodium chloride (Carlo Erba, Milan, Italy), citric acid (Carlo Erba, Milan, Italy) and quinine hydrochloride (Sigma-Aldrich, St Louis, MO, USA) at three levels each were used [27]. The panelists were informed about the taste of each basic concentration. Then, a 10 mL quantity of high and low concentration for each taste solution was served blind. The panelists rinsed their mouths with filtered, de-ionized water between tests. De-ionized water was also used to prepare two blanks. Totaling ten samples (taste solutions and blanks) were presented in random order. The panelists had to identify the intensity (low and high) of each taste solution. The inability to recognize eight out of the 10 taste solutions was used as cutoff point for selection purposes [27]. Afterwards, panelists were trained for the scale use [28].

#### 2.4.2. Quantitative Descriptive Sensory Analysis

A quantitative descriptive analysis (QDA) method [29] was used to assess the sensory profile of the sausages. During preliminary sessions, the panelists were asked to taste some slices of the samples and, on the basis of the available literature [27,30,31], they were encouraged by the panel leader to describe their taste, odor, flavor, appearance and texture and to develop and agree on a consensus list of 22 attributes and their definitions (Table 2).

Standard reference products specific to each identified attribute were administered, with two points of the scale anchored to the reference material. In particular, assessors were repeatedly exposed to the reference samples (three times), indicating the corresponding intensity levels. Subsequently, panelists re-assessed the two levels of intensity of each attribute in blind conditions. Tests were performed in a controlled sensory analysis laboratory [32], equipped with individual booths, under red lighting to mask color differences in the samples, except during the evaluation of appearance, carried out in white fluorescent lighting conditions. For each sample, two 0.4 mm slices (one for appearance, and one for odor/flavor and texture) were obtained using a commercial slicing machine, and immediately served to the panelists at room temperature (20–23 °C). Each sample, coded with three-digit randomized numbers, was served in random order and evaluated in triplicate. For each daily session, five samples were presented. Assessors had to drink a sip of still water at the beginning of the sensory evaluation and to eat unsalted crackers between samples to try to cancel the sensations caused by the previous sample. Attributes were rated on the basis of 100 mm unstructured lines with anchor points at each end (0 = absent, 100 = very strong).

#### 2.4.3. Consumer Testing

Seventy-eight consumers (average age 31 yr.s; 48% men and 52% women) participated in the test. Consumers were recruited among regular eaters of sausages (i.e., consuming the product at least once a week). Under white fluorescent lighting, each consumer evaluated three 0.4 cm thick slices of each sausage in the same controlled sensory analysis laboratory described for QDA. The samples were presented in a random order. Consumers had to drink a sip of still water at the beginning of the sensory evaluation and to eat unsalted crackers between samples to try to cancel the sensations caused by the previous sample. For each product, they expressed an overall liking and a liking for appearance, odor, flavor and texture. Consumers rated their liking on a 9-point hedonic scale, with “extremely unpleasant” (1) at the left end and “extremely pleasant” (9) at the right end [33,34].

The panelists and all consumers were informed about publication of the study.

### 2.5. Statistical Analysis

One-way analysis of variance was used to test the effect of the product affecting color parameters and chemical composition. To identify differences between products the value of least significant difference (LSD) was calculated. Data on batch weights and pH were analyzed using a mixed procedure with product (ten levels) as non-repeated factor and ripening time (four levels) as repeated factor. Sensory profile data were subjected to ANOVA with product (ten levels = two pork/wild boar ratios x five flavor combinations), assessor (ten levels), replicate (three levels) and their first order interactions as factors. Data on consumer test were analyzed by ANOVA with product (ten levels), gender (two levels), age (three levels = 18–39, 40–59, over 60 yr.) and their first order interactions as factors. Principal component analysis (PCA) was performed on the sensory profile and on pH and color parameters to study the relationship between sensory attributes and these parameters. Data were analyzed by R software [35].

## 3. Results

### 3.1. Color Characteristics

All the color parameters, depicted in Table 3, were significantly affected by product. Lightness (*p* < 0.0001) and redness (*p* < 0.0001) was higher in 30 and 30-CF products, while 50-PGW salami showed lower L* value and 30-PW, 30-P and 50-PW had lower a* index. As for b* parameter (*p* = 0.025), 30-CF had the higher value and 50-PW the lower one.

### 3.2. Quantitative Descriptive Sensory Analysis

The ANOVA showed that there were no significant interactions between product × replication or product × assessor, thus indicating that both the training program and the reference frame used were appropriate to reach high reliability of the panel, as the products were consistently evaluated both in different replications and by different assessors.

Product significantly affected the perception of almost all sensory attributes, determining an appreciable differentiation in the different salamis (Table A4). In fact, as for appearance parameters, 50-CF sample was perceived with the highest meat color intensity (73.57 ± 2.69, *p* < 0.0001) and the lowest brightness (28.80 ± 2.66, *p* < 0.0001), while 30 salami showed the opposite intensities for the parameters considered (35.10 ± 2.69 and 51.37 ± 2.66, *p* < 0.0001, for meat color and brightness intensities, respectively). These two products differed markedly also for fennel and hot pepper odor, with the highest intensities in the 50-CF salami (63.13 ± 2.34 and 48.53 ± 2.02, *p* < 0.0001, for fennel and hot pepper odor, respectively) and the lowest in the 30 sample (3.73 ± 2.34 and 3.13 ± 2.02, *p* < 0.0001, for fennel and hot pepper odor, respectively).

As for flavor attributes, the 30-PGW salami tended to show the highest overall intensity (44.17 ± 3.15, *p* = 0.068), especially when compared to the 30 salami (22.97 ± 5.28, *p* = 0.068). Fennel and hot pepper flavor perception confirmed what was found for odor, as the 50-CF salami was perceived with the highest intensities (64.47 ± 3.14 and 48.9 ± 2.60, *p* < 0.001, for fennel and hot pepper flavor, respectively) and the 30 sample with the lowest fennel flavor (4.90 ± 3.14, *p* < 0.0001) while the 50 sample showed the lowest hot pepper flavor (5.37 ± 2.60, *p* < 0.0001). This latest product was also perceived with the highest wild flavor intensity (23.57 ± 2.68, *p* = 0.001); on the contrary, the lowest wild flavor intensity was perceived in the 30-PW salami (7.83 ± 2.76, *p* = 0.001). Again, the 50-CF product had the highest intensity for spiced flavor (65.70 ± 2.97, *p* < 0.001) while the 50 salami showed the lowest intensity (11.07 ± 2.97, *p* < 0.0001).

As for the texture profile, 50-CF sample showed the highest intensities for tenderness, cohesiveness and chewiness (57.90 ± 3.05, *p* = 0.023, 69.67 ± 2.27 and 67.63 ± 2.70, *p* < 0.0001, respectively) while 30 salami had the lowest values for these parameters (40.53 ± 3.05, *p* = 0.023, 47.57 ± 2.27 and 44.73 ± 2.70, *p* < 0.0001, respectively).

**Figure 1:**
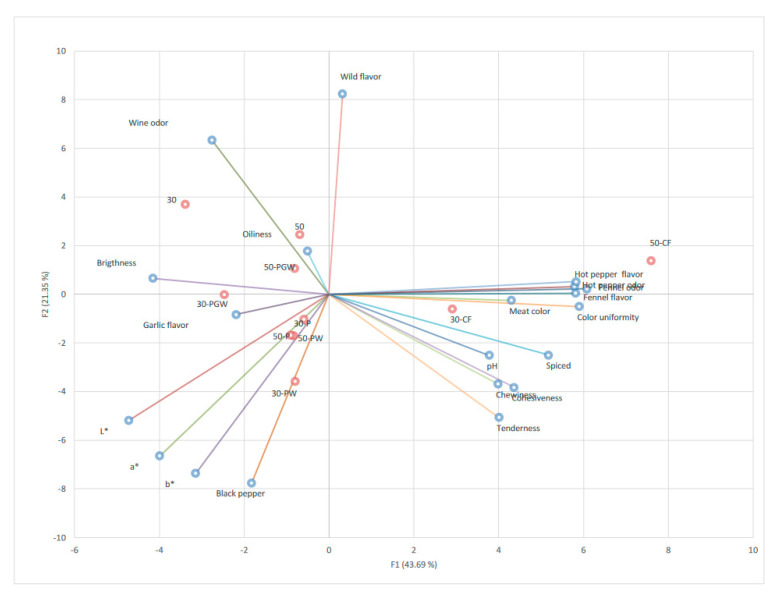
Principal component analysis (PCA).

The PCA bi-plot of the Cacciatore type salami sensory profile, salami pH and color parameters (Figure 1) provided a multivariate graphical representation of the product space showing the relationship between the sensory attributes and the other variables. The first two principal components of PCA explained 65.04% of the variance in the data (46.69% for PC1 and 21.35% for PC2, respectively). In particular, pH (0.58), tenderness (0.62), cohesiveness (0.67), chewiness (0.62), hot pepper flavor (0.90) and odor (0.90), fennel flavor (0.90) and odor (0.94), spiced (0.80), meat color (0.67) and color uniformity (0.91) showed positive correlations with PC1, whereas color parameters, such as L* (-0.73), a* (-0.62), b* (-0.49), brightness (-0.64), wine odor (-0.43), garlic flavor (-0.34), and black pepper flavor (-0.28) were negatively correlated with this axis. On the contrary, PC2 showed positive correlations with wine odor (0.69), and wild flavor (0.89) and negative correlations with spiced (-0.27), chewiness (-0.40), cohesiveness (-0.41), and tenderness (-0.55). This allows a marked characterization of the salamis based on the first component. Products with added chili powder and fennel seeds (30-CF and 50-CF) were differentiated by the other products and located on the right side. On the second component, salamis without flavorings (codes 30 and 50) could be discriminated by those flavored with aromatized garlic wine (30-PGW and 50-PW) or with black pepper only (30-P and 50-P).

### 3.3. Consumer Testing

The results of hedonic testing on 78 consumers are depicted in Table 4. Almost all the products were rated at scores above the neutral point (5 = neither pleasant nor unpleasant), showing that the tested products were perceived as being characterized by a good eating quality. Gender significantly affected all the consumer liking parameters considered, with men attributing the higher scores to the product. Looking at the age groups, appearance (*p* = 0.045), flavor (*p* = 0.002), and texture (*p* = 0.041) of the product were significantly rated best by the youngest consumers.

Product factor significantly affected all the liking parameters. In particular, the sausages from the two batches with chili powder and fennel seeds (30-CF and 50-CF) received the highest scores for overall liking, appearance and odor (*p* = 0.001), and flavor (*p* = 0.031) and texture (*p* = 0.040). In contrast, the sausages that only contained black pepper (30-P and 50-P) were rated worst (Table 5).

## 4. Discussions

Significant differences were found between products for color parameters. This result may be ascribed to the different formulations used in the manufacturing of the ten salamis. In particular, higher percentages of pork meat used in 30 and 30CF salamis may have produced their higher lightness, while the lower value of L* index in 50-PGW product may be due to higher percentage of wild boar meat together with garlic/red wine addition. As for processed meat, other authors [36] found darker color (lower L* values) in wild boar hams compared with Yorkshire hams. Marchiori and de Felicio [37] reported a darker coloration in the wild boar meat compared with pork. Game meat, in fact, has a typical dark red color due to a higher concentration of myoglobin as a result of the intense physical activity of wild animals [38].

The QDA method allowed us to significantly characterize the different products. In agreement with Brankovic Lazic et al. [39], the ingredients used might have significantly affected sensory properties of cacciatore salami. In fact, the 50-CF sample, added with larger quantities of hot pepper powder and fennel seeds, stood out among other products and in particular from sample 30, for highest meat color intensity, fennel and hot pepper odor and flavor intensities, and for textural properties. Probably, hot pepper powder and wild boar meat produced higher meat color intensities.

The sensory characterization of the products may be highlighted even more by PCA graphical representation, where 30-CF and 50-CF salamis stand out from the others on the first component. In addition, similar to stretched curd [40], pH was positively related to some textural attributes, such as cohesiveness, tenderness, and chewiness, whereas a negative correlation was found between pH and oiliness. Despite the fact that we are comparing two extremely different products in the raw material and in the transformation process, in dry-cured fermented sausages textural properties have been mainly related to pH, as stated by Gimeno et al. [41], explaining the variability of texture among different brands of Chorizo de Pamplona. In fact, pH evolution during the ripening process strongly affects the changes in textural attributes [42] and if the pH falls below its isoelectric point, a firmer product is obtained [43]. As for color parameters (L*, a*, b*), they were negatively related with brightness on the second component.

In the consumer test, the products with chili powder and fennel seeds achieved higher liking scores compared with black pepper salami. On this regard, it is worth noting that in Basilicata, the region where the experiment was conducted, many typical cured meats are made with these flavors, so the consumers’ familiarity with these products may have influenced their evaluations. The addition of wild boar meat did not affect consumer liking. Products significantly differed for all the liking parameters. As reported in previous studies on sausages and on hams [44,45], taste is the most important factor affecting purchasing and consumption of dry-cured products, followed by appearance and texture.

A significant gender effect for all the liking parameters was observed, contrastingly with what was reported by Razmaitè et al. [46] for traditional sausages in Lithuania. Although we did not evaluate the effect of information on consumer liking for wild boar products, other studies [10,12,47,48] showed a mostly positive attitude toward wild game products compared to women. In addition, age affected appearance, flavor, and texture liking, with higher scores from the younger consumers. Again, Razmaitè et al. [46] did not find any effect of age on sausages liking, except for the innovations with reduced salt content that were most favorably accepted by the middle-age generation. In addition, Czarniecka-Skubina et al. [13] found that consumers aged 30–40 yr. with higher education and income readily accepted game meat and agreed to eat it in the future. On the contrary, other authors [12,47,49] reported that young people have a more negative attitude towards wild game meat. The positive response to mixed salami could be an opportunity to also drastically reduce the carbon footprint and land use in the production of a widely used and appreciated product such as salami. In our case with a 50% mixed salami, we could halve the carbon footprint and land use values, which for pork are equivalent to 5 kgCO_2_-eq kg^−1^ meat and 40–75 m^2^ yr. kg^−1^ protein, respectively [50].

## 5. Conclusions

Despite the increasing occurrence of wild boar, comparatively few studies deal with quality aspects of meat products of this species. The results of our study, indicating the predominantly satisfactory results of both panel and consumer tests, contribute to a better use of the resource dry-cured wild boar meat. Furthermore, our results show that consumer ratings were influenced by the flavors used, but not by the ratio of wild boar to pork. This opens up the possibility of producing more cost-effective products, as doughs with a high proportion of wild boar meat, in our case 50%, can be used without affecting the acceptability of the product. The development of a wild game meat supply chain could be an effective strategy for the supply of a sustainable alternative to production on intensive livestock farms, for the development of rural territories, and for controlling the growth of wild animal populations. The next studies will look at the variability associated with the effects of hunting methods and field dressing procedures as factors influencing the microbiological quality of the meat and consequently the sensory characteristics of the products made from it. In addition, it would be interesting to investigate the effect of information on the use of wild boar meat in the manufacturing of sausages on consumer liking.

## Figures and Tables

**Table 1:** Ingredients utilized in the ten batches.

Batch	Proportions of Wild Boar Meat/Pork Shoulder/Pork Belly	Ground Black Pepper	Black Pepper Grains	Hot Pepper Powder	Fennel Seeds	Wine	Garlic Flavored Wine
		g/kg				ml/kg	
30	30/40/30	-	-	-	-	-	-
30-PGW		1	4	-	-	-	30
30-PW		1	4	-	-	30	-
30-P		1	4	-	-	-	-
30-CF		-	-	5	2	-	-
50	50/20/30	-	-	-	-	-	-
50-PGW		1	4	-	-	-	30
50-PW		1	4	-	-	30	-
50-P		1	4	-	-	-	-
50-CF		-	-	5	2	-	-

**Table 2:** List of attributes and reference frame used by 10-member panel for Cacciatore salami sensory profiling.

Attributes		Definition	Intensity	
			Low (<20)	High (>80)
Appearance	Color uniformity	Presence of a darker external halo in the slice due to an anomalous drying process	Two-month-seasoned sausage	Bresaola
	Meat color	Intensity of the characteristic red color of the lean of the sausage	Red orange = 2.5 YR ^1^	Dark red = 10 RP ^1^
	Fat color	Intensity of fat color	White = 10 Y ^1^	Pink = 10 R ^1^
	Brightness	Intensity of the characteristic red color (dark-light) of the cured sausage	White	Black
	Fat percentage	Percentage of fat on the slice surface	Bresaola	Hungarian salami
	Fat diameter	Mincing type of fat in the slice	Hungarian salami	Soppressata salami
	Exudate	Amount of liquid fat on the surface	Seasoned sausage	Cacciatore salami
Odor	Overall odor	Level of overall odor before eating the sample	Fifteen-day-seasoned sausage	Napoli salami
	Fennel	Odor associated with fennel seed	Cacciatore salami	Lucanian sausage
	Wine	Odor associated with red wine	Water	Red wine
Flavor	Overall flavor	Level of overall flavor	Fifteen-day-seasoned sausage	Napoli salami
	Fennel	Flavor associated with fennel seed	Low	High
	Black pepper	Flavor associated with the presence of sweet pepper powder	Lucanian sausage	Napoli salami
	Hot pepper	Flavor associated with hot pepper	Seasoned sausage	Lucanian sausage with hot pepper
	Spiced Wine	Flavor associated with mixed aromatic spices Flavor associated with red wine	Seasoned sausage Water	Hungarian salami Red wine
	Wild	Characteristic odor of seasoned wild boar meat	Seasoned sausage	Wild boar sausage
	Garlic	Flavor associated with garlic	Low	High
Texture	Tenderness	Effort required to bite thorough lean and to make the sample ready to be swallowed	Two-month-seasoned cubed sausage	Cubed Hungarian salami
	Cohesiveness	Mechanical textural attribute relating to the degree to which sausage can be deformed before it breaks	Cubed cooked ham	Dry sausage
	Chewiness	Number of chews until reaching a state ready for swallowing	Cubed cooked ham	Dry cured ham
	Oiliness	Perception of the amount of fat released by the product during mastication	Cubed dry cured ham	Cubed Pancetta

^1^ Color definitions as in Munsell Book of Color (X Rite color. Europe GmbH).

**Table 3:** Color parameters of the ten products (mean ± SE) ^(1)^.

Batch	L*	a*	b*
30	42.69 ± 1.23 ^aA^	28.03 ± 0.76 ^aA^	31.48 ± 2.36 ^a^
30-PGW	39.34 ± 1.23 ^bA^	25.73 ± 0.76 ^B^	29.59 ± 2.36 ^A^
30-PW	37.87 ± 1.23 ^aAB^	23.64 ± 0.76 ^B^	29.10 ± 2.36
30-P	35.70 ± 1.23 ^aB^	23.31 ± 0.76 ^B^	29.14 ± 2.36 ^A^
30-CF	41.71 ± 1.23 ^bA^	30.7 ± 0.76 ^bA^	39.7 ± 2.36 ^bB^
50	36.47 ± 1.23 ^B^	25.28 ± 0.76 ^B^	34.06 ± 2.36
50-PGW	34.56 ± 1.23 ^aB^	26.07 ± 0.76 ^bB^	30.20 ± 2.36
50-PW	36.80 ± 1.23 ^B^	23.53 ± 0.76 ^B^	27.12 ± 2.36
50-P	35.19 ± 1.23 ^B^	27.66 ± 0.76 ^B^	32.99 ± 2.36
50-CF	37.38 ± 1.23 ^B^	27.32 ± 0.76 ^B^	33.25 ± 2.36
*p*	<0.0001	<0.0001	0.025

^(1)^ A, B = *p* < 0.01; a, b: *p* < 0.05.

**Table 4:** Hedonic test of Cacciatore salami: effect of gender and age (mean ± SE) ^(1)^.

Liking	Gender		*p*	Class of Age ^(1)^			*p*
	F	M		I	II	III	
Overall	6.11 ± 0.14 ^A^	6.59 ± 0.11 ^B^	0.002	6.46 ± 0.06	6.24 ± 0.17	6.37 ± 0.26	0.484
Appearance	6.12 ± 0.13 ^A^	6.46 ± 0.11 ^B^	0.005	6.54 ± 0.06	6.13 ± 0.17	6.19 ± 0.26	0.045
Odor	6.08 ± 0.13 ^a^	6.27 ± 0.11 ^b^	0.037	6.36 ± 0.06	6.13 ± 0.16	5.94 ± 0.25	0.146
Flavor	6.01 ± 0.15 ^a^	6.30 ± 0.13 ^b^	0.023	6.34 ± 0.07 ^a^	5.93 ± 0.18 ^b^	6.18 ± 0.29	0.002
Texture	6.03 ± 0.13 ^A^	6.56 ± 0.11 ^B^	<0.0001	6.51 ± 0.06 ^a^	6.07 ± 0.17 ^b^	6.31 ± 0.26	0.041

^(1)^ A, B = *p* < 0.01; a, b *p* < 0.05. I = 18–39 yr.; II = 50–59 yr.; III = >60 yr.

**Table 5:** Hedonic test of Cacciatore salami: effect of products (mean ± SE) ^(1)^.

Batch	Liking				
	Overall Liking	Appearance	Odor	Flavor	Texture
30	6.69 ± 0.36 ^a^	6.62 ± 0.35 ^A^	6.48 ± 0.34 ^a^	5.93 ± 0.39 ^b^	6.21 ± 0.35 ^Bb^
30-PGW	6.32 ± 0.36	6.11 ± 0.35 ^b^	6.36 ± 0.34 ^a^	6.07 ± 0.39 ^b^	6.25 ± 0.35
30-PW	6.47 ± 0.36	6.45 ± 0.35 ^a^	6.21 ± 0.34 ^b^	6.25 ± 0.39	6.24 ± 0.35
30-P	5.65 ± 0.36 ^Bb^	5.84 ± 0.35 ^B^	5.45 ± 0.34 ^B^	5.54 ± 0.39 ^B^	5.81 ± 0.35 ^Bb^
30-CF	7.35 ± 0.36 ^A^	7.25 ± 0.35 ^Aa^	7.27 ± 0.34 ^Aa^	7.11 ± 0.39 ^Aa^	7.18 ± 0.35 ^Aa^
50	6.24 ± 0.36	6.74 ± 0.35 ^A^	6.12 ± 0.34 ^b^	6.22 ± 0.39	6.36 ± 0.35
50-PGW	6.02 ± 0.36 ^Bb^	5.78 ± 0.35 ^B^	5.54 ± 0.34 ^B^	5.85 ± 0.39 ^B^	6.10 ± 0.35 ^B^
50-PW	6.69 ± 0.36 ^a^	6.60 ± 0.35 ^A^	6.33 ± 0.34 ^a^	6.53 ± 0.39	6.60 ± 0.35
50-P	5.73 ± 0.36 ^B^	5.57 ± 0.35 ^B^	5.54 ± 0.34 ^B^	5.61 ± 0.39 ^B^	5.82 ± 0.35 ^B^
50-CF	7.07 ± 0.36 ^Aa^	6.98 ± 0.35 ^A^	6.95 ± 0.34 ^A^	7.03 ± 0.39 ^A^	7.04 ± 0.35 ^Aa^
*p*	0.001	0.001	0.001	0.031	0.040

^(1)^ A, B = *p* < 0.01; a, b *p* < 0.05.

## Data Availability

Data are available on request.

## Appendix A

**Table A1:** Average values of the batch weights (kg) of sausages during drying and curing, and total weight loss (%) (mean ± SE).

Batch	Days of Ripening												Total Weight Loss, 0–30 d
	0			10			20			30			
30	2.70	±	0.10	1.66	±	0.16	1.58	±	0.13	1.49	±	0.11	44.81
30-PGW	2.71	±	0.21	1.74	±	0.12	1.65	±	0.11	1.57	±	0.10	42.07
30-PW	2.75	±	0.40	1.72	±	0.29	1.64	±	0.27	1.57	±	0.25	42.91
30-P	2.71	±	0.41	1.78	±	0.24	1.71	±	0.20	1.64	±	0.16	39.48
30-CF	2.59	±	0.49	1.75	±	0.40	1.67	±	0.38	1.60	±	0.36	38.22
50	2.70	±	0.36	1.69	±	0.26	1.60	±	0.24	1.51	±	0.21	44.07
50-PGW	2.62	±	0.24	1.70	±	0.30	1.58	±	0.23	1.45	±	0.24	44.66
50-PW	2.84	±	0.11	1.73	±	0.07	1.64	±	0.06	1.54	±	0.05	45.77
50-P	2.73	±	0.04	1.76	±	0.05	1.67	±	0.03	1.57	±	0.01	42.49
50-CF	2.72	±	0.18	1.61	±	0.11	1.54	±	0.11	1.47	±	0.11	45.96
*p*	0.056			0.184			0.078			0.265			

**Table A2:** pH values on day 2, 4, 6 and 30 of ripening (mean ± SE) ^(1)^.

Batch	Days of Ripening											
	2			4			6			30		
30	5.79	±	0.08	5.76	±	0.12	5.87 a	±	0.09	5.63 ^a^	±	0.04
30-PGW	5.66	±	0.05	5.46	±	0.05	5.51	±	0.03	5.96	±	0.05
30-PW	5.63	±	0.87	5.42	±	0.08	5.54	±	0.12	5.92	±	0.09
30-P	5.57	±	0.11	5.58	±	0.1	5.58	±	0.04	6.17	±	0.87
30-CF	5.68	±	0.04	5.48	±	0.08	5.69	±	0.06	6.19 ^b^	±	0.07
50	5.71	±	0.03	5.64	±	0.05	5.77	±	0.09	5.85	±	0.08
50-PGW	5.61	±	0.06	5.40	±	0.05	5.24 b	±	0.13	5.81	±	0.12
50-PW	5.60	±	0.05	5.44	±	0.05	5.58	±	0.04	5.75	±	0.05
50-P	5.56	±	0.07	5.41	±	0.03	5.53	±	0.12	5.94	±	0.10
50-CF	5.74	±	0.07	5.52	±	0.1	5.81	±	0.08	5.74	±	0.08
*p*	0.078			0.227			0.033			0.041		

^(1)^ a, b = *p* < 0.05.

**Table A3:** Proximate chemical composition of the products at the end of ripening (mean ± SE) ^(1)^.

Batch	Dry Matter			Collagen			Connective			Total Protein			Fat			NaCl			Ash		
30	67.94 ^a^	±	1.97	2.47	±	0.07	8.71 ^a^	±	0.25	28.34 a	±	0.82	30.61 ^a^	±	0.89	3.79	±	0.11	8.03	±	0.23
30-PGW	66.03	±	1.91	2.62	±	0.08	9.23	±	0.27	30.03	±	0.87	32.42	±	0.94	4.02	±	0.12	8.51	±	0.25
30-PW	66.31	±	1.92	2.6	±	0.08	9.15	±	0.27	29.78	±	0.86	32.15	±	0.93	3.98	±	0.12	8.44	±	0.24
30-P	64.86	±	1.88	2.71	±	0.08	9.55 ^b^	±	0.28	31.06	±	0.90	33.54 ^b^	±	0.97	4.15	±	0.12	8.08	±	0.26
30-CF	64.78 ^b^	±	1.88	2.71	±	0.08	9.57 ^b^	±	0.28	31.14 b	±	0.90	33.62 ^b^	±	0.97	4.16	±	0.12	8.82	±	0.26
50	66.69	±	1.93	2.57	±	0.07	9.05	±	0.26	29.44	±	0.85	31.79	±	0.92	3.94	±	0.11	8.34	±	0.24
50-PGW	66.89	±	1.94	2.55	±	0.07	9.00	±	0.26	29.27	±	0.85	31.61	±	0.92	3.91	±	0.11	8.29	±	0.24
50-PW	67.25	±	1.95	2.52	±	0.07	8.90	±	0.26	28.95	±	0.84	31.26	±	0.91	3.87	±	0.11	8.20	±	0.24
50-P	66.17	±	1.92	2.61	±	0.08	9.19	±	0.27	29.90	±	0.87	32.29	±	0.94	4.00	±	0.12	8.47	±	0.25
50-CF	67.31	±	1.95	2.52	±	0.07	8.88	±	0.26	28.90	±	0.84	31.20	±	0.90	3.86	±	0.11	8.19	±	0.24
*p*	0.044			0.349			0.023			0.028			0.029			0.088			0.213		

^(1)^ a, b = *p* < 0.05.

**Table A4:** Sensory profile, as assessed by a 10-member trained panel, of Cacciatore salami (mean ± SE) ^(1)^

Attribute	Batch										*p*
	30	30-PGW	30-PW	30-P	30-CF	50	50-PGW	50-PW	50-P	50-CF	
	Appearance										
Color uniformity	29.70 ± 2.76 ^B^	29.80 ± 2.76 ^B^	37.22 ± 2.83	33.40 ± 2.76	39.40 ± 2.76 ^a^	33.37 ± 2.76 ^B^	39.07 ± 2.76 ^a^	33.83 ± 7.76 ^B^	34.57 ± 2.76	47.50 ± 2.76 ^Aa^	0.001
Meat color	35.10 ± 2.69 ^Ba^	38.53 ± 2.76 ^B^	47.83 ± 2.76 ^A^	37.47 ± 2.69 ^B^	41.00± 2.69 ^B^	46.77 ± 2.69 ^A^	61.33 ± 2.6 ^A^	56.93 ± 2.69 ^A^	51.27 ± 2.69 ^A^	73.57 ± 2.69 ^A^	<0.0001
Fat color	54.37 ± 3.37	53.97 ± 3.37	52.54 ± 3.47	60.30 ± 3.37 ^Aa^	47.30 ± 3.37 ^B^	54.47 ± 3.37	55.17 ± 3.37	48.77 ± 3.37 ^b^	48.47 ± 3.37 ^b^	54.30 ± 3.37	0.227
Brightness	51.37 ± 2.66 ^A^	49.47 ± 2.66	45.71 ± 2.73 ^Aa^	47.43 ± 2.66 ^A^	45.83 ± 2.66	47.40 ± 2.66	33.87 ± 2.66 ^B^	39.37 ± 2.66 ^bB^	36.43 ± 2.66 ^Bb^	28.80 ± 2.66 ^B^	<0.0001
Fat percentage	58.63 ± 2.25 ^Aa^	57.97 ± 2.25 ^A^	55.97 ± 2.31	58.67 ± 2.25 ^A^	53.23 ± 2.25	52.70 ± 2.25	49.10 ± 2.25 ^Bb^	56.43 ± 2.25	56.53 ± 2.25	44.67 ± 2.25 ^B^	0.002
Fat diameter	44.27 ± 2.87 ^A^	50.03 ± 2.87 ^Aa^	36.54 ± 2.94 ^B^	38.00 ± 2.87 ^B^	37.00 ± 2.87 ^B^	44.07 ± 2.87 ^A^	49.20 ± 2.87 ^A^	45.73 ± 2.87	38.0 ± 2.87 ^b^	44.37 ± 2.87 ^B^	0.002
Exudate	39.40 ± 3.46	34.00 ± 3.46 ^B^	48.62 ± 3.55 ^Aa^	38.30 ± 3.46	39.77 ± 3.46	37.80 ± 3.46	38.96 ± 3.46	45.03 ± 3.46 ^A^	36.83 ± 3.46 ^ab^	34.23 ± 3.46 ^B^	0.102
	Odor										
Overall odor	22.97 ± 5.28 ^B^	33.20 ± 5.28 ^B^	38.24 ± 5.43	41.47 ± 5.28 ^A^	39.17 ± 5.28 ^Aa^	27.97 ± 5.28 ^b^	31.07 ± 5.28	34.03 ± 5.28	25.86 ± 5.28 ^B^	31.77 ± 5.28 ^B^	0.258
Fennel	3.73 ± 2.34 ^B^	10.30 ± 2.34 ^A^	14.94 ± 2.40 ^a^	13.40 ± 2.34 ^B^	49.67 ± 2.34 ^C^	6.17 ± 2.34 ^B^	9.60 ± 2.34	6.73 ± 2.34 ^b^	5.63 ± 2.34	63.13 ± 2.34	<0.0001
Wine	20.33 ± 2.82	18.27 ± 2.82	7.68±2.90 ^B^	15.43 ± 2.82	12.70 ± 2.82	14.77 ± 2.82	21.43 ± 2.82 ^A^	13.20 ± 2.82 ^B^	13.20 ± 2.82 ^B^	12.47 ± 2.82 ^B^	0.033
Garlic	14.97 ± 2.75	14.87 ± 2.75	10.89 ± 2.82 ^B^	10.17 ± 2.75 ^Bb^	8.63 ± 2.75 ^B^	12.97 ± 2.75	18.37 ± 2.75 ^Aa^	17.73 ± 2.75 ^A^	12.20 ± 2.75	16.03 ± 2.75 ^A^	0.195
Red hot pepp.	3.13 ± 2.02 ^B^	3.73 ±2.02 ^B^	7.13±2.08	7.80 ± 2.02 ^B^	47.30 ± 2.02 ^A^	2.73 ± 2.02 ^B^	6.50 ± 2.02 ^B^	5.10 ± 2.02 ^B^	4.43 ± 2.02 ^B^	48.53 ± 2.02 ^A^	<0.0001
	Taste										
Bitter	20.90 ± 3.43 ^B^	19.00 ± 3.43	20.61 ± 3.53	23.90 ± 3.43	16.80 ± 3.43 ^B^	21.87 ± 3.43	20.27 ± 3.43	30.00± 3.43 ^A^	27.20 ± 3.43 ^A^	22.57 ± 3.43	0.249
	Flavor										
Overall flavor	29.77 ± 3.15 ^Bb^	44.17 ± 3.15 ^A^	40.42 ± 3.24	39.70 ± 3.15 ^A^	36.37 ± 3.15	32.47 ± 3.15	36.13 ± 3.15	38.63 ± 3.15 ^Aa^	35.40 ± 3.15	33.17 ± 3.15 ^a^	0.068
Fennel	4.90 ± 3.14 ^Bb^	11.40 ± 3.14 ^B^	12.41 ± 3.22 ^B^	15.57 ± 3.14 ^Ba^	63.20 ± 3.14 ^A^	5.43 ± 3.14 ^B^	9.43 ± 3.14 ^B^	8.23 ± 3.14 ^B^	9.40 ± 3.14 ^B^	64.47 ± 3.14 ^A^	<0.0001
Red hot pepp.	6.23 ± 2.60 ^B^	9.27 ± 2.60	8.89 ± 2.60	9.10 ± 2.60 ^B^	46.50 ± 2.60 ^A^	5.37 ± 2.60 ^B^	9.00 ± 2.60	6.67 ± 2.60 ^B^	8.57 ± 2.60 ^B^	48.90 ± 2.60 ^A^	<0.0001
Black pepper	14.13 ± 3.62 ^Ca^	41.43 ±3.62	72.99 ± 3.62 ^A^	69.33 ± 3.62 ^A^	19.77± 3.62 ^a^	4.27 ± 3.62 ^Bb^	29.17 ± 3.62	42.03 ± 3.62 ^C^	65.43 ± 3.62 ^A^	15.30 ± 3.62 ^a^	<0.0001
Wild	22.33 ± 2.68 ^A^	13.63 ± 2.68	7.83 ± 2.76 ^B^	10.90 ± 2.68	10.70 ± 2.68	23.57 ± 2.68 ^Aa^	19.03 ± 2.68 ^A^	14.97 ± 2.68 ^b^	14.53 ± 2.68 ^b^	20.50 ± 2.68 ^A^	0.001
Spiced	23.57 ± 2.97	24.87 ± 2.97 ^D^	41.54 ± 3.05	40.00 ± 2.97	63.53 ± 2.97 ^A^	11.07 ± 2.97 ^Bb^	20.57 ± 2.97 ^Da^	21.50 ±2.97	35.47 ± 2.97 ^C^	65.70 ± 2.97 ^A^	<0.0001
Garlic	9.53 ± 2.77 ^B^	15.50 ± 2.77	9.66 ± 2.84 ^B^	10.13± 2.77	6.83 ± 2.77 ^Bb^	10.53 ± 2.77	20.06 ± 2.77 ^A^	15.93 ± 2.77 ^a^	14.83 ± 2.77	10.20 ± 2.77	0.030
Wine	14.83 ± 3.00	18.73 ± 3.00	15.06 ± 3.07	21.10 ± 3.00 ^a^	12.33± 3.00	13.90 ± 3.00	22.90 ± 3.00 ^A^	18.13 ± 3.00	17.03 ± 3.00	10.97 ± 3.00 ^Bb^	*0.116*
	Texture										
Tenderness	40.53 ± 3.05 ^Bb^	50.73 ± 3.05	53.21 ± 3.13 ^A^	55.30 ± 3.05 ^A^	52.33 ± 3.05 ^A^	52.63 ± 3.05 ^A^	52.93 ± 3.05 ^A^	53.97 ± 3.05 ^A^	51.57 ± 3.05 ^a^	57.90 ± 3.05 ^A^	*0.023*
Cohesiveness	47.57 ± 2.27 ^B^	56.53 ± 2.27 ^C^	59.77 ±2.34 ^C^	61.97 ± 2.27 ^b^	57.93 ± 2.27 ^C^	60.33 ± 2.27 C	61.27 ± 2.27	65.07 ± 2.27 ^A^	61.70 ± 2.27 ^b^	69.67 ± 2.27 ^Aa^	*<0.0001*
Chewiness	44.73 ± 2.70 ^B^	57.83 ± 2.70 ^B^	58.56 ± 2.77 ^B^	61.10 ± 2.70 ^B^	55.43 ± 2.70 ^B^	57.80 ± 2.70 ^B^	61.37 ± 2.70 ^B^	62.23 ± 2.70 ^B^	58.13± 2.70 ^B^	67.63 ± 2.70 ^B^	*<0.0001*
Oiliness	44.60 ± 5.24 ^b^	44.60 ± 5.24 ^B^	46.91 ± 5.38 ^B^	51.87 ± 5.24 ^b^	49.43 ± 5.24 ^b^	53.20 ± 5.24	67.03 ± 5.24 ^Aa^	56.93 ± 5.24	49.50 ± 5.24	48.97 ± 5.24	*0.170*

^(1)^ a, b, = *p* < 0.05; A, B, C, D = *p* < 0.01.
